# Integrative deep learning analysis of 2D and 3D body composition features for predicting postoperative pancreatic fistula after distal pancreatectomy

**DOI:** 10.1080/07853890.2025.2597067

**Published:** 2025-12-04

**Authors:** Keke Liang, Qi Miao, Yi Jing, Jingao Xu, He Zhang, Kexin Zhu, Yashu Liu, Ruimei Chai, Guoguang Fan, Xiaodong Tan

**Affiliations:** ^a^ Department of General Surgery, Shengjing Hospital of China Medical University, Shenyang, China; ^b^ Department of Radiology, First Hospital of China Medical University, Shenyang, China; ^c^ Neusoft Research of Intelligent Healthcare Technology, Co. Ltd, Shenyang, China

**Keywords:** Body composition, postoperative pancreatic fistula, distal pancreatectomy, artificial intelligence, computed tomography

## Abstract

**Background:**

Accurate prediction of postoperative pancreatic fistula (POPF) after radical distal pancreatectomy (DP) is critical for optimizing surgical strategies. The objective of this study was to develop and validate a deep learning-based framework for three-dimensional (3D) body composition analysis to forecast POPF in patients with pancreatic cancer (PC).

**Methods:**

A retrospective analysis was conducted on patients who underwent radical DP at two institutions between 2015 and 2022. A deep learning-based 2D and 3D segmentation model was developed to assess abdominal muscles and fat using preoperative computed tomography (CT) images. Predictive models for POPF were constructed using Gradient Boosting Decision Trees (GBDT) and their performance was evaluated using the area under the receiver operating characteristic curve (AUC) and decision curve analysis (DCA) on validation and external test datasets.

**Results:**

The study comprised 230 patients with PC, with a mean age of 62 years. The incidence of POPF was observed to be 47.4% (Grade B/C). In terms of muscle segmentation, the Dice similarity coefficients (DSCs) for the testing set varied between 91.84 and 98.41% across different regions of the abdominal musculature. For the segmentation of visceral and subcutaneous adipose tissue, the DSCs in the testing set were 97.10 and 98.57%, respectively. The integrated clinical and imaging-based POPF prediction model demonstrated superior performance, achieving an AUC of 0.82, with a sensitivity of 0.81 and a specificity of 0.76 in the external test set.

**Conclusion:**

The implementation of a deep learning-based 2D and 3D body composition analysis pipeline exhibited high accuracy in predicting POPF following radical DP for PC. However, further validation in larger, multicenter cohorts is required to confirm the generalizability of these findings.

## Introduction

Pancreatic cancer (PC) is a highly malignant cancer, with a five-year survival rate of only about 13% [1]. Radical distal pancreatectomy (DP) is the standard procedure for removing malignant tumors located in the body or tail of pancreas [2]. With the improvement of surgical techniques, the perioperative mortality of DP decreased significantly, less than 5% [3]. However, the incidence of postoperative complications after distal pancreatectomy is still high [4].

Postoperative pancreatic fistula (POPF) remains the most common complication after DP and occurs in 5 ∼ 64% [5–8]. The development of POPF can increase the risk of postoperative hemorrhage, infection, delayed gastric emptying, prolonged hospital stay, and even death [5,9]. Predicting POPF after DP is crucial for improving surgical planning, patient counseling, and risk management, ultimately reducing morbidity and mortality associated with this complication [6,10].

The prediction of POPF following DP has prompted the development of various models utilizing clinical and imaging features. In the DISPAIR fistula risk score model, three variables (transection site of pancreas, pancreatic thickness at transection site, and diabetes) were significantly associated with POPF after DP and the area under the receiver operating characteristic curve (AUC) was 0.798 (0.748–0.848) [11]. The pre-operative DP fistula risk score (D-FRS) model included 2 variables (pancreatic thickness and pancreatic duct diameter) and achieved an AUC of 0.73 [12]. However, these models include both malignant and benign pancreatic tumors. The surgical approach and scope vary greatly depending on the nature and location of tumor. For malignant tumors located in pancreatic body or tail the surgical method is generally standardized, involving the resection of the pancreas at the neck and performing a radical DP with splenectomy [2]. On the other hand, the surgical approach for benign tumors is less consistent. The pancreas is usually removed from the tumor’s right side, albeit the precise site may change. In additional, the decision to preserve the spleen may also be considered [13]. Literature indicates that the site of pancreatic transection can influence the incidence of POPF following DP [11]. Therefore, constructing a POPF prediction model specifically for patients with PC located in the pancreatic body and tail may enhance the applicability and accuracy of the prediction model.

Imaging-derived body composition analysis has emerged as a significant tool in predicting tumor prognosis and surgical complications. Multiple studies have shown that a low skeletal muscle index (SMI) has been consistently associated with poorer prognosis in various malignancies, including colorectal, lung, and pancreatic cancers [14–16]. Our previous study also demonstrated that myosteatosis is significantly associated with both survival outcomes and postoperative complications in patients with PC [17]. Furthermore, in the study by Pecorelli et al. visceral fat was shown to be associated with POPF following DP [18]. However, most of these models rely on image analysis focused on a single axial level, commonly the third lumbar (L3) vertebra. Although this method offers an estimation, it fails to accurately represent patients’ unique body composition. In additional, the lack of automation limits its practicality for routine clinical use. A recent study demonstrated that 3D volumetric body composition parameters are more strongly associated with colorectal cancer outcomes than coventional two-dimensional (2D) parameters [19].

At present, there is a lack of published research examining the potential of 3D body composition indices to enhance the prediction of POPF in patients with pancreatic body and tail cancer. Consequently, this study aimed to develop a 3D automatic segmentation model for abdominal muscles and fat using preoperative computed tomography (CT) images, and to utilize deep learning-based body composition segmentation for the prediction of POPF following radical DP for PC.

## Methods

### Study population

**Figure 1.**
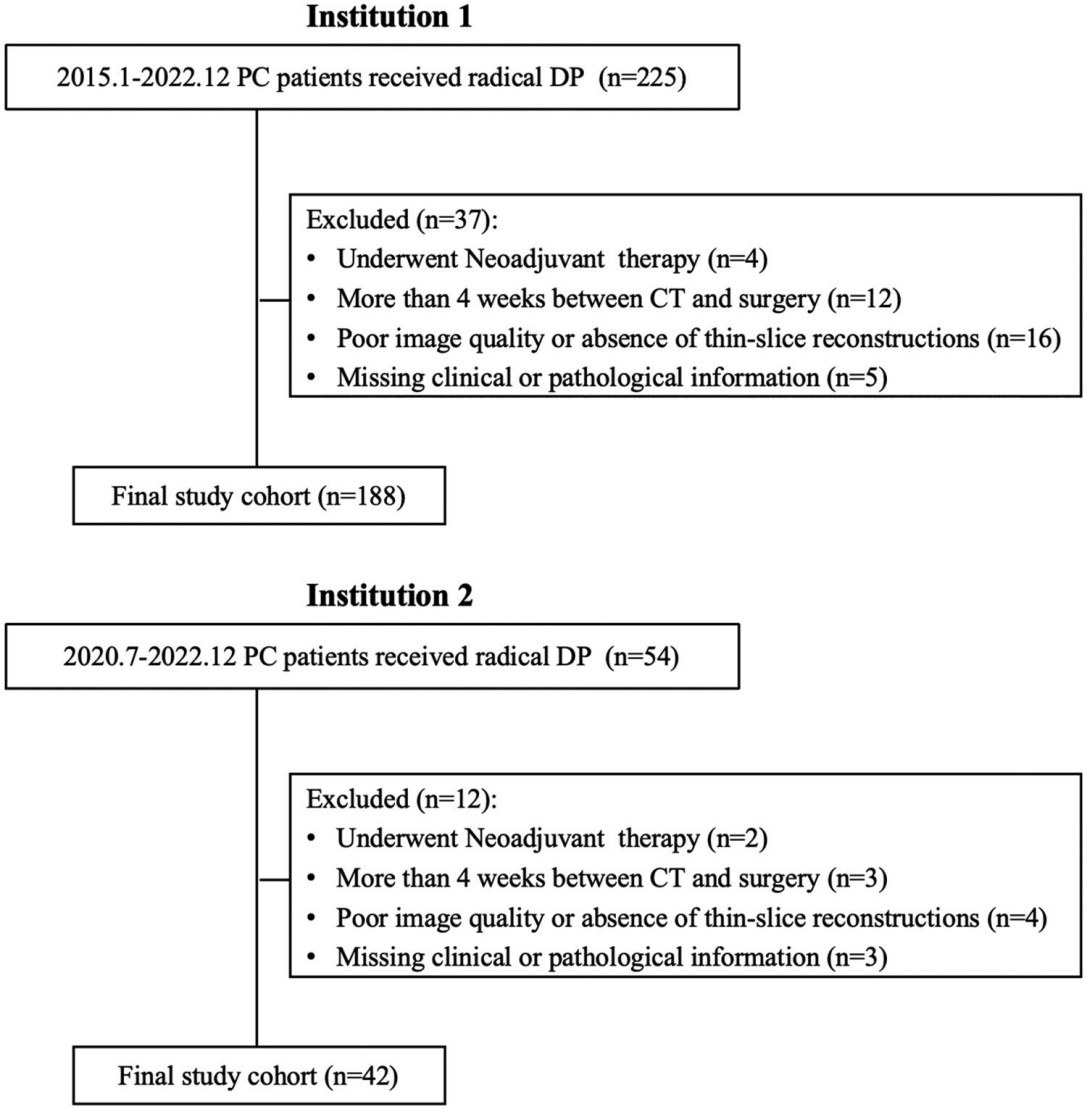
The recruitment workflow of PC patients at two institutions.

This retrospective study cohort comprised consecutive patients who underwent radical DP with histopathologically confirmed PC at two institutions: Shengjing Hospital of China Medical University (from January 2015 to December 2022) and The First Hospital of China Medical University (from July 2020 to December 2022). Inclusion criteria were: (1) radical DP with pathological confirmation of PC, and (2) preoperative contrast-enhanced abdominal CT performed. Exclusion criteria included: (1) preoperative radiotherapy or chemotherapy, (2) more than a 4-week interval between CT and surgery, (3) inadequate CT quality (e.g. missing thin-slice reconstructions or severe artifacts), and (4) incomplete clinical or pathological data. The selection process of study subjects is illustrated in Figure 1. The patients from Shengjing Hospital were divided into a training set and a validation set at a 9:1 ratio, while the patients from The First Hospital served as the external test set (Supplementary Figure 1).

### Data collection

Demographic, clinical, and laboratory data—including sex, age, height, weight, complete blood count, prealbumin, albumin and pancreatic transection method—were obtained from medical records. All laboratory data were collected at the preoperative baseline within two weeks prior to surgery. Based on the above data, several nutritional, inflammatory, and immune indices were calculated as follows: Body mass index (BMI)=wight(kg)/height^2^(m^2^), Prognostic nutritional index (PNI)=albumin(g/L)+5 × lymphocyte count(10^9^/L), Neutrophil-to-lymphocyte ratio (NLR)=neutrophil count(10^9^/L)/lymphocyte count(10^9^/L), Platelet-to-lymphocyte ratio (PLR)=platelet count(10^9^/L)/lymphocyte count(10^9^/L), Lymphocyte-to-monocyte ratio (LMR)=lymphocyte count(10^9^/L)/monocyte count(10^9^/L), and Systemic immune-inflammation index (SII)=platelet count(10^9^/L) × NLR.

All CT scans were acquired using 64-detector CT systems. Scanning parameters are detailed in our previously study [20]. The portal venous phase (PVP) images were used for subsequent analyses. Two senior abdominal radiologists (M.Q. and C.RM.) who were blinded to clinical and histopathological information measured tumor size, pancreatic neck thickness, and pancreatic duct diameter at the neck. When the pancreatic duct was not visible or measured less than 1 mm on CT, it was uniformly recorded as 1 mm. They also manually segmented muscle and adipose tissue in CT images from the first lumbar (L1) vertebra to the L3 vertebra of 49 PC cases randomly selected from Institution 1 using 3D Slicer (version 4.13.0). The muscle segmentation was divided into four distinct parts: the rectus abdominis, lateral abdominal muscles (including the external oblique, internal oblique, and transversus abdominis), posterior abdominal muscles (erector spinae and quadratus lumborum), and psoas major. The adipose tissue segmentation was carried out by separately segmenting subcutaneous fat, visceral fat, and intermuscular fat.

POPF was defined according to the 2016 International Study Group on Pancreatic Fistula guidelines, with Grade B/C considered positive and Grade A negative [21].

### Deep learning-based 3D segmentation of body composition

**Figure 2.**
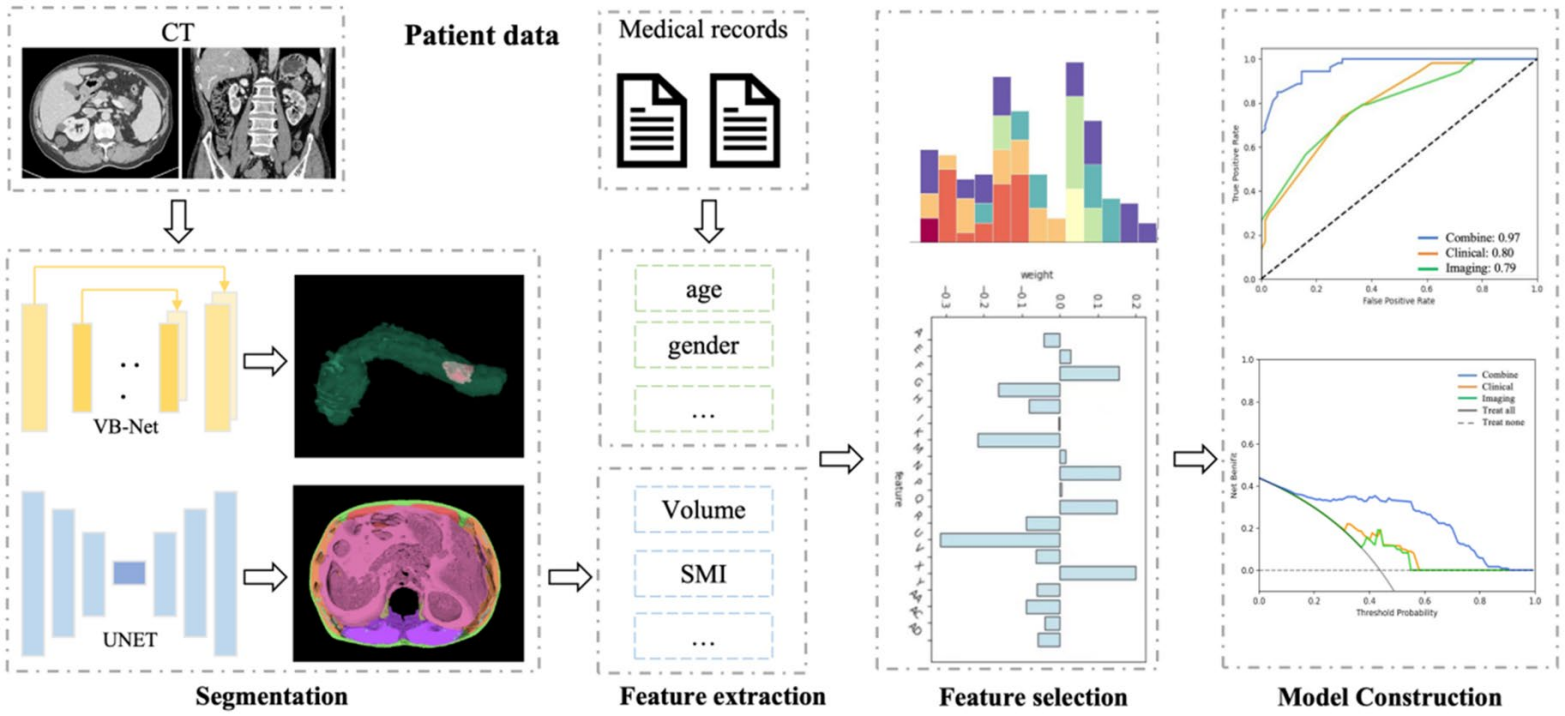
The flowchart of the automatic pipeline for 3D body composition segmentation and POPF prediction for PC patients following radical DP.

Figure 2 illustrates the flowchart of the automated pipeline designed for body composition segmentation and POPF prediction in PC patients. We employed advanced automated 3D segmentation methods to define regions of interest (ROIs) for the abdominal muscular and adipose tissues, spanning from the upper border of L1 vertebra to the lower border of the L3 vertebra. Pancreas and pancreatic tumor 3D segmentation leveraged a previously validated model [20], while abdominal muscle and adipose tissue segmentation used a new nnU-Net–based model [22].

The muscle and adipose segmentation model employed a U-shaped architecture with 18 convolutional blocks (3 × 3 × 3 kernel, Instance Normalization, Leaky ReLU), as illustrated in Supplementary Figure 2. Downsampling used pooling factors of 2 × 2 × 2 for the first three stages and 1 × 2 × 2 for the final stage; the corresponding upsampling kernel sizes mirrored these patterns. Deep supervision was integrated to improve accuracy. With a batch size of 2, SGD optimizer (learning rate 0.01, weight decay 3e-5), and a total of 400 epochs, training was performed on a 32GB V100 GPU (Ubuntu 20.04.1 LTS, PyTorch 1.11.0). The 49 annotated cases selected from Institution 1 were split into training, validation, and testing sets at a 7:2:1 ratio. Each patient’s CT scan contains approximately 500 slices.

### Feature extraction and selection

Following segmentation, we extracted both 2D features from the single L3 level and 3D features-mean CT values, volumes, and mean areas from the L1-L3 ROIs, ultimately deriving 58 imaging features detailed in Supplementary Table 1. In additional, 19 clinically relevant indicators that may be associated with POPF were collected and was shown in Table 1.

**Table 1.** Characteristics of PC patients used for POPF prediction.

Variable	All (*n* = 230)	Training set (*n* = 169)	Validation set (*n* = 19)	External test set (*n* = 42)	*P* value^#^
Sex					0.566
Male(%)	115(50.0)	81(47.9)	10(52.6)	24(57.1)	
Female(%)	115(50.0)	88(52.1)	9(47.4)	18(42.9)	
Age(years)	62.0 ± 0.6	61.9 ± 0.6	62.0 ± 2.4	62.1 ± 1.5	0.990
*BMI(kg/m²)	22.6(21.2–24.3)	22.6(21.2–24.3)	22.8(20.5–24.2)	22.6(21.2–24.7)	0.997
*WBC(10^^^9/L)	5.7(4.7–6.8)	5.8(4.8–7.0)	5.4(5.0–6.3)	5.2(4.5–6.4)	0.264
*Neutrophils(10^^^9/L)	3.5(2.7–4.3)	3.6(2.7–4.56)	3.2(2.6–3.8)	3.4(2.8–3.7)	0.155
*Lymphocytes(10^^^9/L)	1.5(1.2–1.9)	1.5(1.2–1.9)	1.7(1.5–2.0)	1.6(1.2–2.0)	0.361
*Monocytes(10^^^9/L)	0.4(0.3–0.5)	0.4(0.3–0.5)	0.5(0.3–0.6)	0.3(0.3–0.5)	0.283
*Platelet(10^^^9/L)	182.0(148.0–224.0)	179.0(143.0–235.0)	192.0(162.0–227.0)	192.0(155.0–220.0)	0.584
*Prealbumin(g/L)	0.22(0.18–0.25)	0.22(0.18–0.25)	0.24(0.18–0.26)	0.22(0.18–0.25)	0.407
*Albumin(g/L)	41.5(39.2–43.3)	41.5(39.3–43.9)	41.9(38.8–44.0)	41.2(39.2–42.5)	0.208
*PNI	49.1(46.2–52.0)	49.0(46.0–52.0)	49.8(47.9–53.3)	49.0(46.5–51.6)	0.720
*NLR	2.3(1.7–3.3)	2.4(1.8–3.4)	1.9(1.5–2.5)	2.0(1.5–3.1)	0.053
*PLR	122.1(99.5–145.7)	122.1(101.3–145.9)	110.3(92.4–140.9)	122.7 (93.2–150.6)	0.845
*LMR	4.0(2.9–5.2)	4.0(2.8–5.2)	3.6(3.0–5.0)	4.4(3.1–5.3)	0.212
*SII	419.8(285.3–585.9)	446.8(291.6–624.5)	352.0(273.5–552.2)	347.2(264.1–543.8)	0.117
*Tumor size(cm)	3.6 (2.8–4.8)	3.8 (3.0–5.0)	4.0 (2.9–5.4)	3.0 (2.0–3.9)	0.005
Pancreatic neck thickness(mm)	11.2 ± 0.3	11.5 ± 0.3	11.2 ± 1.0	10.0 ± 0.6	0.063
*Diameter of pancreatic duct at neck (mm)	1.0 (1.0-1.0)	1.0(1.0-1.0)	1.0(1.0-1.0)	1.0(1.0-1.0)	0.467
Transection of pancreatic neck(%)					
Stapler	155 (67.4)	109(64.5)	10 (52.6)	36 (85.7)	0.010
Manual	75 (32.6)	60 (35.5)	9 (47.4)	6 (14.3)	
POPF(%)					0.552
Yes	109 (47.4)	77 (45.6)	11 (57.9)	21 (50.0)	
No	121 (52.6)	92 (54.4)	8 (42.1)	21 (50.0)	

*Median with interquartile range.

^#^*P*-value represent comparisons among the training, validation, and external test sets.

Feature selection was performed using Recursive Feature Elimination (RFE) with a Decision Tree base model. Before RFE, all data were standardized using Z-scores. The optimal number of retained features was determined by maximizing cross-validated accuracy. The final subset of features was then used for model construction.

### Machine learning model construction for POPF prediction

We employed a Gradient Boosting Decision Tree (GBDT) algorithm—implemented *via* the Python scikit-learn package (version 1.3.2)—to develop each of the three prediction models (Model_Clinical_, Model_Imaging_ and Model_Combine_). GBDT is an ensemble learning method in which multiple shallow decision trees are trained sequentially: at each iteration the model fits a new tree to the negative gradient (residuals) of the current loss, thereby progressively improving the prediction function. This approach offers strong capability for modelling non-linear relationships and interactions, is comparatively robust to missing values and outliers, and does not require extensive feature scaling or transformation.

For hyper-parameter optimisation, we conducted a grid search on the training cohort and evaluated candidate models on the validation cohort. The search space was defined as: n_estimators = 5 to 50 (step = 5); max_depth = 2 to 7 (step = 1); min_samples_split = 2 to 6 (step = 1); and the loss function was set to ‘log_loss’ for binary classification. The optimal parameter set selected was: n_estimators = 10, max_depth = 2, min_samples_split = 2, and loss = ‘log_loss’. We then retrained the final model on the combined training + validation set using these parameters and assessed performance on the independent test set. The relatively shallow tree depth and limited number of estimators were chosen to mitigate over-fitting given our modest sample size, while preserving interpretability.

Finally, we leveraged GBDT’s built-in feature importance metrics to assess each feature’s impact on prediction accuracy, thereby providing insights into key predictors of POPF.

### Statistical analysis

Data analysis was performed utilizing R software (version 4.1.1, R Foundation for Statistical Computing). For continuous variables, data were described using the mean and standard deviation (SD) for normally distributed data, and the median and interquartile range (IQR) for non-normally distributed data. Comparisons of continuous variables were conducted using either the independent sample *t*-test or the Mann-Whitney *U* test, contingent upon the distribution of the data. Categorical variables were analyzed *via* the Chi-square test. Segmentation performance was quantified using the Dice similarity coefficients (DSCs). The discriminatory capacity of the models was assessed through receiver operating characteristic (ROC) curves, with the receiver operating characteristic curve (AUC) and its 95% confidence interval (CI) calculated to evaluate model efficacy. The Youden index was employed to determine the optimal cutoff value. Decision curve analysis (DCA) was utilized to appraise clinical utility and net benefit. Model comparisons were executed using the DeLong test. A *P*-value of less than 0.05 was considered indicative of statistical significance.

## Results

### Patient characteristics

The flowchart of patient recruitment is shown in Figure 1. A total of 230 patients were included. Among them, males and females each accounted for 50%, with a mean age of 62 years. The mean pancreatic neck thickness was 11.2 mm, and the median pancreatic duct diameter at the neck was 1 mm. The overall incidence of POPF was 47.4%. In external test set, patients exhibited smaller tumor diameters compared to those in training and validation sets. In additional, the proportion of patients undergoing pancreatic transection using staplers during surgery was higher in the external test cohort. No significant differences were observed in other clinical variables across the groups. Details are presented in Table 1.

### Automated 3D segmentation of abdominal muscular and adipose tissues

**Figure 3.**
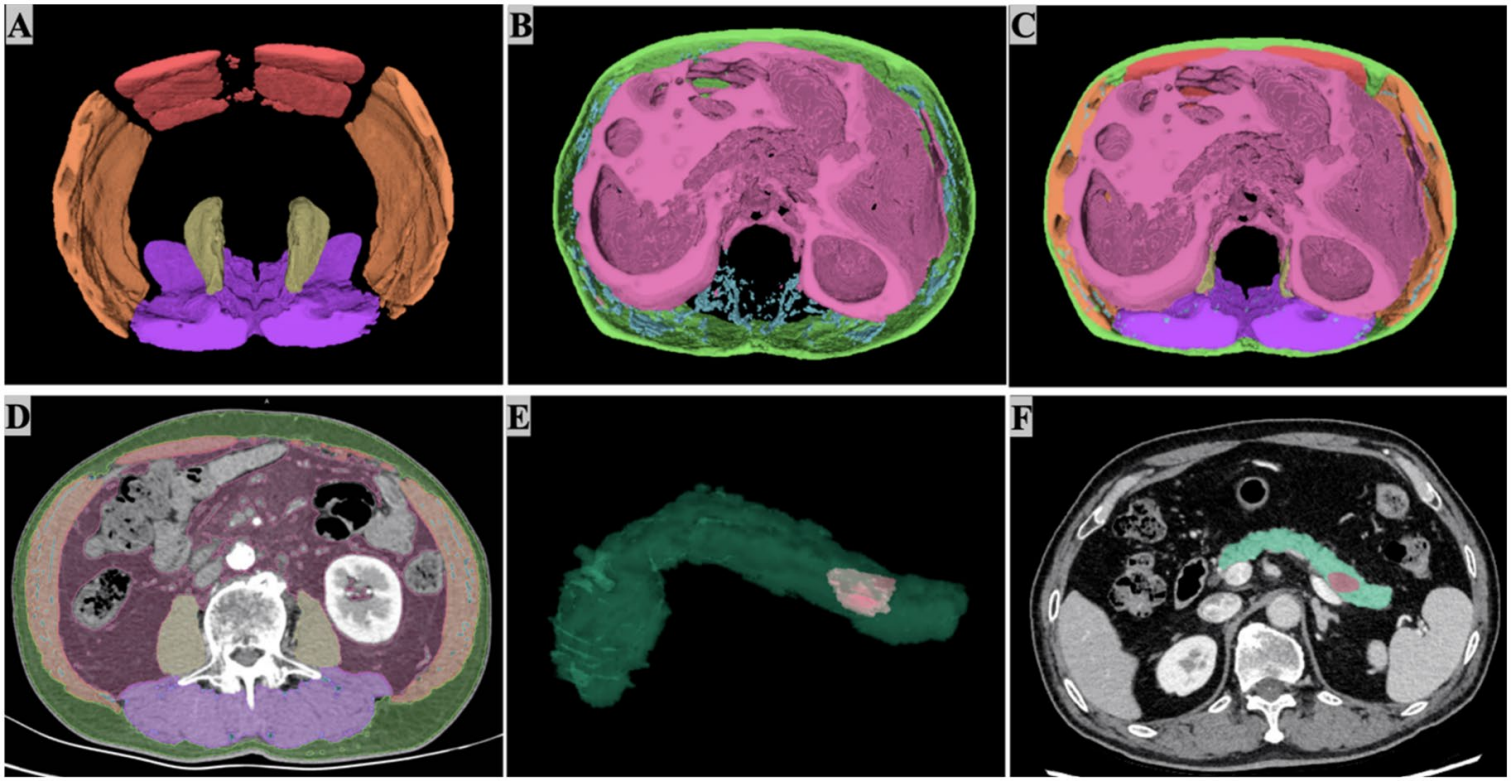
Diagram illustrating the automatic segmentation of body composition, pancreas, and tumor. 3D segmented image of abdominal muscle (A), abdominal fat (B) and both (C) spanning L1 to L3 vertebrae. (D) 2D CT image of abdominal muscle and fat at the mid-layer of the L3 vertebra. (E) 3D segmented image of pancreas and pancreatic tumor. (F) 2D CT image of pancreas and pancreatic tumor. A-D (Red: rectus abdominis; Orange: lateral abdominal muscles; Purple: posterior abdominal muscles; Yellow: psoas major; Green: subcutaneous fat; Blue: intermuscular fat; Pink: visceral fat). E-F (Teal: pancreas; Pink: pancreatic tumor).

The segmentation model was trained, validated, and tested on CT images from 34, 10, and 5 PC patients, respectively, covering the L1 to L3 intervals. The performance on abdominal muscle and adipose tissue segmentation is shown in Figures 3A–D and Table 2. For muscle segmentation, the DSCs in the testing set ranged from 91.84 to 98.41% in different regions of abdominal muscle. Regarding visceral and subcutaneous fat segmentation, the DSCs in the testing set were 97.10 and 98.57%, respectively. However, for intermuscular fat, the DSC is 78.04%. The segmentation results of the pancreas and pancreatic tumors using our previously developed segmentation model are shown in Figure 3E, F.

**Table 2.** DSCs For abdominal composition segmentation.

Segmented region	DSCs	
	Validation set	Test set
Rectus abdominis	94.88%	91.84%
Lateral abdominal muscles	95.78%	93.61%
Posterior abdominal muscles	98.31%	98.41%
Psoas major	96.67%	97.13%
Intermuscular fat	78.40%	78.04%
Visceral fat	96.48%	97.10%
Subcutaneous fat	98.45%	98.57%

### Predicting POPF following radical DP for PC

Through rigorous feature selection, ten optimal predictors were identified for inclusion in our model: five clinical features - platelet-to-lymphocyte ratio (PLR), prognostic nutritional index (PNI), lymphocyte count, white blood cell count, and prealbumin; along with five CT-derived imaging features—tumor-to-pancreas density ratio (DR_tumor/pancreas_), tumor-to-pancreas volume ratio (VR_tumor/pancreas_), L3-level subcutaneous fat index (SFI_L3_), intermuscular-to-subcutaneous fat density ratio between L1-L3 (DR_intermuscular fat/subcutaneous fat_), and L3 subcutaneous fat CT attenuation (D_subcutaneous fat(L3)_) (Table 3). The selected clinical features are primarily related to the patients’ nutritional and inflammatory status, reflecting their systemic health conditions. For the imaging features, three are focused on subcutaneous fat parameters, while the other two are associated with tumor-to-pancreas ratios, emphasizing their relevance to anatomical and structural characteristics.

**Table 3.** Clinical and imaging features retained after feature selection.

	Feature name				
Clinical features	PLR	PNI	Lymphocyte count	White blood cell count	Prealbumin
Imaging features	DR_tumor/pancreas_	VR_tumor/pancreas_	SFI_L3_	DR_intermuscular fat/subcutaneous fat(L1-L3)_	D_subcutaneous fat(L3)_

PLR: platelet-to-lymphocyte ratio; PNI: prognostic nutritional index; DR_tumor/pancreas:_ ratio of mean CT value of entire tumor to that of entire pancreas; VR_tumor/pancreas:_ ratio of tumor volume to pancreatic volume; SFI_L3:_cross-sectional area of subcutaneous fat at the mid-level of L3 / height^2^; DR_intermuscular fat/subcutaneous fat(L1-L3):_ ratio of mean CT value of intermuscular fat to that of subcutaneous fat between L1 and L3; D_subcutaneous fat(L3):_ CT value of subcutaneous fat at the mid-level of L3.

**Figure 4.**
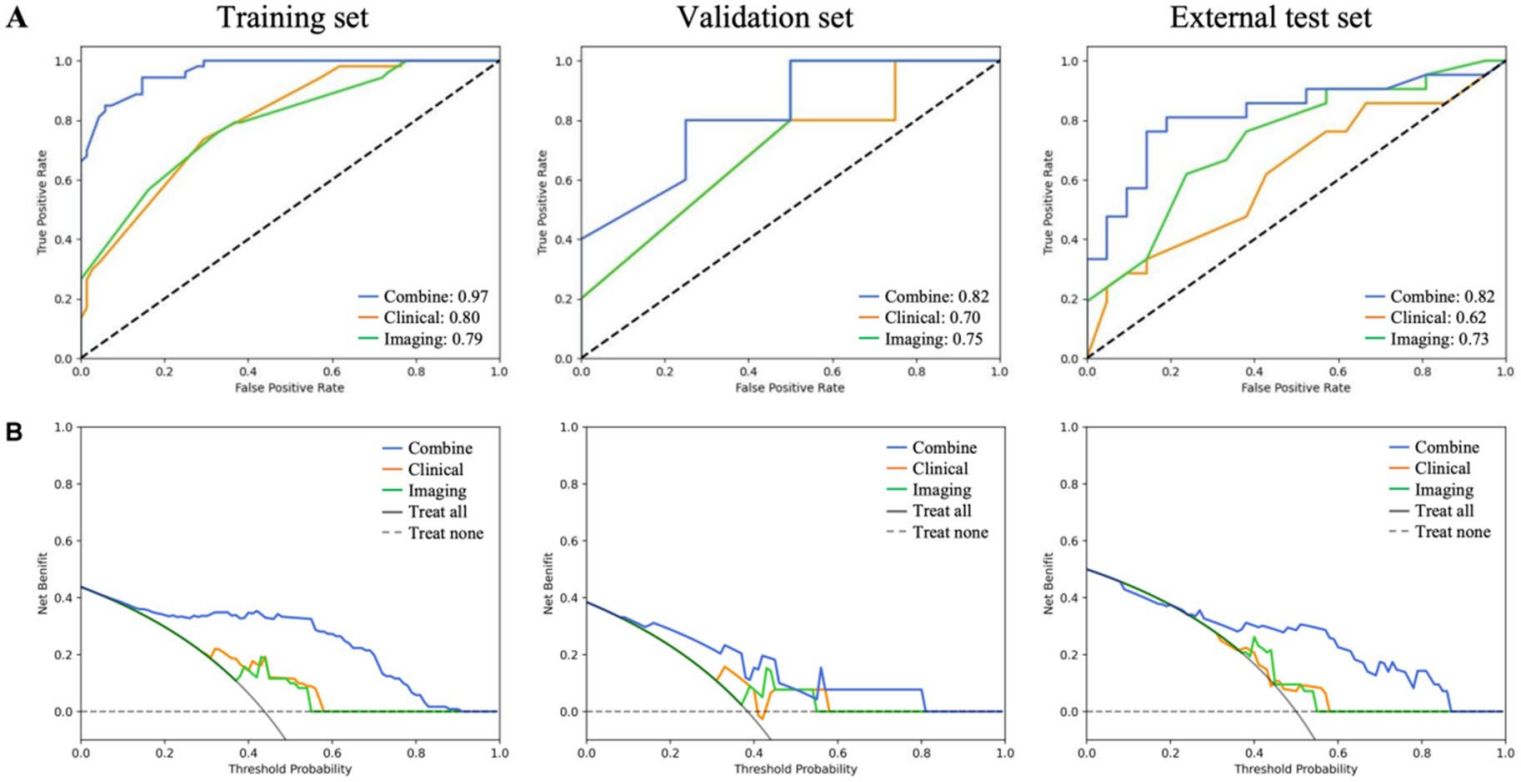
The performance of three models (Model_Combine_, Model_Clinical_, and Model_Imaging_) in predicting POPF. (A) ROCs of three models in training set, validation set and external test set. (B) DCAs of three models in training set, validation set and external test set.

The predictive performance of the three models is summarized in Table 4 and Supplementary Table 2. In the external test set, Model_Combine_ achieved an AUC of 0.82 (95% CI: 0.67–0.94), with a sensitivity of 0.81, specificity of 0.76, and accuracy of 0.79 for predicting POPF. It’s AUC was significantly higher than that of Model_Clinical_ (AUC:0.62, *P* = 0.003), but the difference compared with the Model_Imaging_ (AUC:0.73) was not statistically significant (*P* = 0.362) (Table 4, Figure 4A). In additional, the DCA curve of these three models in predicting POPF was shown in Figure 4B.

**Table 4.** Predictive performance of AI models for POPF in external test set.

	Sensitivity	Specificity	Accuracy	AUC (95% CI)	*P* Value
Model_Clinical_	0.62	0.57	0.60	0.62 (0.44–0.78)	0.003
Model_Imaging_	0.67	0.67	0.67	0.73 (0.58–0.88)	0.362
Model_Combine_	0.81	0.76	0.79	0.82 (0.67–0.94)	–

*P* Value: Delong test was used to compare the AUCs of Model_Clinical_, Model_Imaging_ with Model_Combine._

### Feature importance analysis

**Figure 5.**
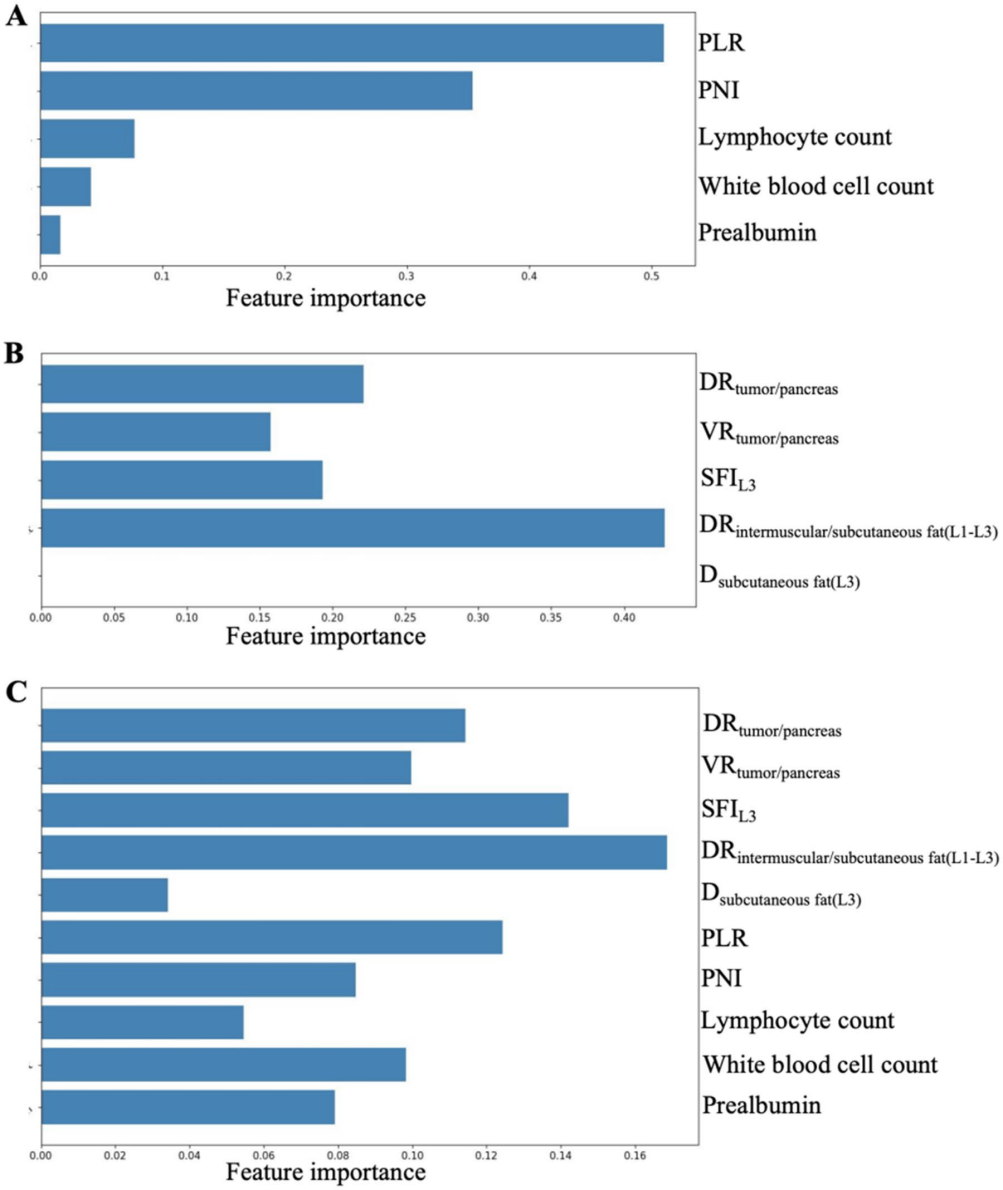
Feature importance ranking for (A) Model_Clinical_, (B) Model_Imaging_, and (C) Model_Combine_, respectively. PLR: platelet-to-lymphocyte ratio; PNI: prognostic nutritional index; DR_tumor/pancreas_: ratio of mean CT value of entire tumor to that of entire pancreas; VR_tumor/pancreas_: ratio of tumor volume to pancreatic volume; SFI_L3_:cross-sectional area of subcutaneous fat at the mid-level of L3 / height^2^; DR_intermuscular fat/subcutaneous fat(L1-L3)_: ratio of mean CT value of intermuscular fat to that of subcutaneous fat between L1 and L3; D_subcutaneous fat(L3)_: CT value of subcutaneous fat at the mid-level of L3.

The feature importance rankings for the three models are presented in Figure 5. PLR and PNI emerged as the most influential predictors in Model_Clinical_. In Model_Imaging_, DR_intermuscular/subcutaneous fat (L1-L3)_ demonstrated the highest importance, followed by DR_tumor/pancreas_ and SFI_L3_. In Model_Combine_, DR_intermuscular/subcutaneous fat (L1-L3)_ remained the most important feature, with SFI_L3_, DR_tumor/pancreas_, and VR_tumor/pancreas_ also playing significant roles. Clinical predictors, including PLR and PNI, also contributed meaningfully to the combined model’s performance, albeit to a lesser extent compared to imaging-derived features.

## Discussion

In this multi-center study, we developed and validated an automated artificial intelligence (AI) pipeline for 2D and 3D body composition segmentation and the prediction of POPF in PC patients following radical DP. Nevertheless, there are several limitations that warrant consideration. Firstly, the sample size was relatively small, which may limit the stability of the model and increase the risk of overfitting. Secondly, although the segmentation model demonstrated high accuracy overall, its performance in segmenting intermuscular fat was somewhat lower (DSC = 78.05%), which may impact the precision of associated features. Thirdly, the different enrollment periods between the two centers may have introduced selection or temporal bias.

By integrating imaging-derived 2D and 3D body composition metrics with clinical features, we constructed a predictive model demonstrating high performance, as indicated by an AUC of 0.82 in the external test set. Our findings underscore the significant role of the automated deep learning-based pipeline, which integrates both 2D and 3D body composition features, in enhancing the predictive accuracy of POPF.

In recent years, body composition has been increasingly recognized as a crucial factor in predicting prognosis, treatment efficacy, and postoperative complications in cancer patients [17,23–25]. Traditionally, body composition analysis has relied on manual segmentation of 2D images at a single axial level, typically the L3 vertebra. This approach has limitations: extremely time-consuming; a single slice cannot represent the 3D variability of body composition; and manual segmentation introduces inter-reader variability, reducing reproducibility [26]. Advances in AI have made 3D automatic segmentation models feasible, addressing these challenges [27]. Our study employed a deep learning-based framework for 3D segmentation from L1-L3 vertebrae. This model achieved high DSCs for muscle (>90%) and adipose tissue (subcutaneous fat: 98.57%, visceral fat: 97.1%) segmentation. The DSC for intermuscular fat was slightly lower (78.04%), which is within the acceptable range reported in previous studies of body composition segmentation [28]. This lower segmentation DSC of intermuscular fat may be possibly due to its minimal volume, irregular shape, and indistinct boundaries with muscles, which also complicate manual gold standard definition. Segmentation variability in this region and small sample size in model construction may introduce potential uncertainty. In addition, differences in CT scanning parameters across centers may also affect the stability and generalizability of the model. Therefore, future studies using larger, multi-center datasets and enhanced deep learning models will be needed to further refine intermuscular fat quantification and enhance model stability.

Existing POPF prediction models often include mixed patient populations comprising both malignant and benign pancreatic tumors, which may introduce variability and limit specificity. In the DISPAIR fistula risk score model, two of the three variables associated with POPF—transection site of the pancreas and pancreatic thickness at the transection site—are directly related to the pancreatic transection location [11]. Among patients with pancreatic body and tail cancer, the pancreatic transection is almost exclusively performed at the pancreatic neck. This is the primary reason why we chose to focus exclusively on patients with pancreatic body and tail cancer in this study. Our model focuses exclusively on PC patients undergoing radical DP, enhancing its specificity to this patient population. This targeted approach achieved an AUC of 0.82 in the external test set, surpassing the performance of previously reported models (AUC range: 0.730–0.798) [11,12,18]. Furthermore, we validated both models in our test cohort of 42 cases and obtained an AUC of 0.561 (0.381–0.742) for the D-FRS model and 0.566 (0.388–0.744) for the DISPAIR model (Supplementary Figure 3). The lower performance may be attributed to differences in the patient population, as our study included only patients with pancreatic cancer. By focusing the cohort exclusively on PC patients, our model reduces confounding variables such as variations in tumor biology and surgical techniques, thereby enhancing its reliability and generalizability for clinical application.

Previous studies have shown that pancreatic thickness and pancreatic duct diameter are two key imaging features associated with POPF [9,11]. However, in our model, among the five features ultimately included, three are related to subcutaneous fat, while the other two are associated with the volume and density of the pancreas and tumor. Our results suggest that abdominal fat is closely related to the occurrence of POPF. Additionally, with the aid of the 3D segmentation model, we identified that beyond traditional imaging features, the ratio of tumor to pancreatic volume and density is also closely related to POPF. At the same time, both 3D imaging features and 2D imaging features (e.g. D_subcutaneous fat (L3)_ and SFI_L3_) were included in our predictive model. This result indicates that, compared to using either 3D or 2D imaging features alone, the combined use of both may achieve better predictive performance. Interestingly, the five clinical features included in the final model are all systemic indicators of nutrition, inflammation, and immunity, suggesting that the occurrence of POPF is not only associated with local surgical factors but also strongly influenced by the overall systemic condition [29]. These findings highlight the critical importance and justification for employing body composition analysis in predicting POPF. Nonetheless, these results require further validation through subsequent large-scale studies.

In the feature importance analysis, four of the top five features in the combined model were derived from imaging, underscoring the essential role of imaging characteristics in developing robust models for predicting POPF. Notably, the top three features included DR_intermuscular fat to subcutaneous fat (L1-L3)_, SFI_L3_, and PLR, emphasizing the significance of intermuscular and subcutaneous fat density at the L1-L3 interval and the subcutaneous fat area at the L3 level in predicting POPF. Previous studies have reported that infiltration of adipose tissue into muscle can lead to insulin resistance and impair immune function in patients with tumors. In addition, with the increase in muscle adipose tissue, the secretion of various proinflammatory factors rises, which can affect systemic inflammation and tumor progression [30]. Systemic inflammation and immune-dysfunction may further predispose patients to POPF by impairing wound healing, reducing microvascular perfusion of the pancreatic stump [31]. Additionally, the tumor-to-pancreas density and volume ratios ranked fourth and fifth, further illustrating the influence of tumor burden on POPF risk. Furthermore, the incorporation of clinical features, such as PLR and PNI, highlights the multifactorial nature of POPF. These markers reflect the systemic inflammatory and nutritional status of patients, suggesting that a combination of imaging and clinical metrics provides a more comprehensive assessment of risk factors.

## Conclusion

This study underscores the utility of deep learning-derived 2D and 3D body composition analysis in predicting POPF following radical DP for PC. Our findings emphasize the importance of integrate 2D and 3D body composition analysis and its potential for personalized risk stratification in PC surgery. Future research should focus on refining and validating these models across more extensive patient cohorts to confirm the generalizability of these findings.

## Supplementary Material

Supplementary Material.docx

## Data Availability

The data that support the findings of this study are available from the corresponding author upon reasonable request.
