# Supplementary material for: Integrative deep learning analysis of 2D and 3D body composition features for predicting postoperative pancreatic fistula after distal pancreatectomy
Source: Ann Med. 2025 Dec 4;57(1):2597067. doi: 10.1080/07853890.2025.2597067 (PMC12679842; doi:10.1080/07853890.2025.2597067)
Supplement: Supplementary Material.docx [file IANN_A_2597067_SM3243.docx]

**Supplementary Material**

**Integrative deep learning analysis of 2D and 3D body composition features for predicting postoperative pancreatic fistula after distal pancreatectomy**

Keke Liang^1,*^, Qi Miao^2,*^, Yi Jing^3^, Jingao Xu^3^, He Zhang^3^, Kexin Zhu^2^, Yashu Liu^1^, Ruimei Chai^2^, Guoguang Fan^2,#^, Xiaodong Tan^1,#^

^1^Department of General Surgery, Shengjing Hospital of China Medical University, Shenyang, China.

^2^Department of Radiology, First Hospital of China Medical University, Shenyang, China.

^3^Neusoft Research of Intelligent Healthcare Technology, Co. Ltd, Shenyang, China.

^*^ These authors contributed equally to this work as co-first authors.

^#^ These authors contributed equally to this work as co-corresponding authors.

**Supplementary Table 1. Extracted clinical features for the prediction of POPF**

| Interval | Indicator | Feature Name | Description |
| --- | --- | --- | --- |
| L1-L3  (from the superior endplate of the first lumbar vertebra to the inferior endplate of the third lumbar vertebra) | CT value  (three-dimension) | D_rectus abdominis(L1-L3)_ | mean CT value (density) of rectus abdominis between L1 and L3 vertebral interval |
|  |  | D_lateral muscles(L1-L3)_ | mean CT value (density) of lateral abdominal muscles between L1 and L3 vertebral interval |
|  |  | D_posterior muscles(L1-L3)_ | mean CT value (density) of posterior abdominal muscles between L1 and L3 vertebral interval |
|  |  | D_psoas major(L1-L3)_ | mean CT value (density) of psoas major between L1 and L3 vertebral interval |
|  |  | D_all muscles(L1-L3)_ | mean CT value (density) of all abdominal muscles between L1 and L3 vertebral interval |
|  |  | D_intermuscular fat(L1-L3)_ | mean CT value (density) of intermuscular fat between L1 and L3 vertebral interval |
|  |  | D_visceral fat(L1-L3)_ | mean CT value (density) of visceral fat between L1 and L3 vertebral interval |
|  |  | D_subcutaneous fat(L1-L3)_ | mean CT value (density) of subcutaneous fat between L1 and L3 vertebral interval |
|  |  | DR_intermuscular fat/all muscles(L1-L3)_ | ratio of mean CT value of intermuscular fat to that of all muscles between L1 and L3 vertebral interval |
|  |  | DR_visceral fat/all muscles(L1-L3)_ | ratio of mean CT value of visceral fat to that of all muscles between L1 and L3 vertebral interval |
|  |  | DR_subcutaneous fat/all muscles(L1-L3)_ | ratio of mean CT value of subcutaneous fat to that of all muscles between L1 and L3 vertebral interval |
|  |  | DR_subcutaneous fat/visceral fat(L1-L3)_ | ratio of mean CT value of subcutaneous fat to that of visceral fat between L1 and L3 vertebral interval |
|  |  | DR_intermuscular fat/subcutaneous fat(L1-L3)_ | ratio of mean CT value of intermuscular fat to that of subcutaneous fat between L1 and L3 vertebral interval |
|  |  | DR_intermuscular fat/visceral fat(L1-L3)_ | ratio of mean CT value of intermuscular fat to that of visceral fat between L1 and L3 vertebral interval |
|  |  | D_tumor_ | mean CT value (density)of entire panceatic tumor |
|  |  | D_pancreas_ | mean CT value (density) of entire pancreas |
|  |  | DR_tumor/pancreas_ | ratio of mean CT value of entire tumor to that of entire pancreas |
|  | Volume  (three-dimension) | V_rectus abdominis(L1-L3)_ | volume of rectus abdominis between L1 and L3 vertebral interval |
|  |  | V_lateral muscles(L1-L3)_ | volume of lateral abdominal muscles between L1 and L3 vertebral interval |
|  |  | V_posterior muscles(L1-L3)_ | volume of posterior abdominal muscles between L1 and L3 vertebral interval |
|  |  | V_psoas major(L1-L3)_ | volume of psoas major between L1 and L3 vertebral interval |
|  |  | V_all muscles(L1-L3)_ | volume of all muscles between L1 and L3 vertebral interval |
|  |  | V_intermuscular fat(L1-L3)_ | volume of intermuscular fat between L1 and L3 vertebral interval |
|  |  | V_visceral fat(L1-L3)_ | volume of visceral fat between L1 and L3 vertebral interval |
|  |  | V_subcutaneous fat(L1-L3)_ | volume of subcutaneous fat between L1 and L3 vertebral interval |
|  |  | VR_intermuscular fat/all muscles(L1-L3)_ | ratio of intermuscular fat volume to all muscle volume between L1 and L3 vertebral interval |
|  |  | VR_visceral fat/all muscles(L1-L3)_ | ratio of visceral fat volume to all muscle volume between L1 and L3 vertebral interval |
|  |  | VR_subcutaneous fat/all muscles(L1-L3)_ | ratio of subcutaneous fat volume to all muscle volume between L1 and L3 vertebral interval |
|  |  | VR_subcutaneous fat/visceral fat(L1-L3)_ | ratio of subcutaneous fat volume to visceral fat volume between L1 and L3 vertebral interval |
|  |  | VR_intermuscular fat/subcutaneous fat(L1-L3)_ | ratio of intermuscular fat volume to subcutaneous fat volume between L1 and L3 vertebral interval |
|  |  | VR_intermuscular fat/visceral fat(L1-L3)_ | ratio of intermuscular fat volume to visceral fat volume between L1 and L3 vertebral interval |
|  |  | V_tumor_ | volume of pancreatic tumor |
|  |  | V_pancreas_ | volume of pancreas |
|  |  | VR_tumor/pancreas_ | ratio of tumor volume to pancreatic volume |
|  | Skeletal muscle index, SMI (three-dimension) | SMI_L1-L3_ | mean skeletal muscle index(SMI) between L1 and L3 vertebral interval= volume of all muscles between L1 and L3 vertebral interval / thickness from L1 to L3 / height^2^ |
|  | Fat index, FI  (three-dimension) | SFI_L1-L3_ | mean subcutaneous fat index(SFI) between L1 and L3 vertebral interval= volume of subcutaneous fat between L1 and L3 vertebral interval / thickness from L1 to L3 / height^2^ |
|  |  | IFI_L1-L3_ | mean intermuscular fat index(IFI) between L1 and L3 vertebral interval= volume of intermuscular fat between L1 and L3 vertebral interval / thickness from L1 to L3 / height^2^ |
|  |  | VFI_L1-L3_ | mean visceral fat index(VFI) between L1 and L3 vertebral interval= volume of visceral fat between L1 and L3 vertebral interval / thickness from L1 to L3 / height^2^ |
| L3  (a single layer at the mid-level of the third lumbar vertebra) | CT value  (two-dimension) | D_rectus abdominis(L3)_ | CT value (density) of rectus abdominis at the mid-level of L3 vertebral |
|  |  | D_lateral muscles(L3)_ | CT value (density) of lateral abdominal muscles at the mid-level of L3 vertebral |
|  |  | D_posterior muscles(L3)_ | CT value (density) of posterior abdominal muscles at the mid-level of L3 vertebral |
|  |  | D_psoas major(L3)_ | CT value (density) of psoas major at the mid-level of L3 vertebral |
|  |  | D_all muscles(L3)_ | CT value (density) of all abdominal muscles at the mid-level of L3 vertebral |
|  |  | D_intermuscular fat(L3)_ | CT value (density) of intermuscular fat at the mid-level of L3 vertebral |
|  |  | D_visceral fat(L3)_ | CT value (density) of visceral fat at the mid-level of L3 vertebral |
|  |  | D_subcutaneous fat(L3)_ | CT value (density) of subcutaneous fat at the mid-level of L3 vertebral |
|  | Area  (two-dimension) | A_rectus abdominis(L3)_ | cross-sectional area of rectus abdominis at the mid-level of L3 vertebral |
|  |  | A_lateral muscles(L3)_ | cross-sectional area of lateral muscles at the mid-level of L3 vertebral |
|  |  | A_posterior muscles(L3)_ | cross-sectional area of posterior muscles at the mid-level of L3 vertebral |
|  |  | A_psoas major(L3)_ | cross-sectional area of psoas major at the mid-level of L3 vertebral |
|  |  | A_all muscles(L3)_ | cross-sectional area of all muscles at the mid-level of L3 vertebral |
|  |  | A_intermuscular fat(L3)_ | cross-sectional area of intermuscular fat at the mid-level of L3 vertebral |
|  |  | A_visceral fat(L3)_ | cross-sectional area of visceral fat at the mid-level of L3 vertebral |
|  |  | A_subcutaneous fat(L3)_ | cross-sectional area of subcutaneous fat at the mid-level of L3 vertebral |
|  | Skeletal muscle index, SMI (two-dimension) | SMI_(L3)_ | skeletal muscle index(SMI) at the mid-level of L3 vertebral = cross-sectional area of all segmented abdominal muscles at the mid-level of L3 vertebral / height^2^ (cm^2^/m^2^) |
|  | Fat index, FI  (two-dimension) | SFI_L3_ | subcutaneous fat index(SFI) at the mid-level of L3 vertebral = cross-sectional area of subcutaneous fat at the mid-level of L3 vertebral / height^2^ (cm^2^/m^2^) |
|  |  | IFI_L3_ | intermuscular fat index(IFI) at the mid-level of L3 vertebral = cross-sectional area of intermuscular fat at the mid-level of L3 vertebral / height^2^ (cm^2^/m^2^) |
|  |  | VFI_L3_ | visceral fat index(VFI) at the mid-level of L3 vertebral = cross-sectional area of visceral fat at the mid-level of L3 vertebral / height^2^ (cm^2^/m^2^) |

**Supplementary Table 2. Predictive performance of AI Models for POPF in training and validation sets.**

|  | Training set (n=169) | | | |  | Validation set (n=19) | | | |  |
| --- | --- | --- | --- | --- | --- | --- | --- | --- | --- | --- |
|  | Sensitivity | Specificity | Accuracy | AUC(95% CI) |  | Sensitivity | Specificity | Accuracy | AUC(95% CI) |  |
| Model_Clinical_ | 0.74 | 0.71 | 0.72 | 0.80(0.72-0.87) |  | 0.80 | 0.50 | 0.62 | 0.70(0.40-0.94) |  |
| Model_Imaging_ | 0.75 | 0.68 | 0.71 | 0.79(0.71-0.87) |  | 0.80 | 0.50 | 0.62 | 0.75(0.51-0.95) |  |
| Model_Combine_ | 0.89 | 0.87 | 0.88 | 0.97(0.94-0.99) |  | 0.80 | 0.75 | 0.77 | 0.82(0.52-1.00) |  |


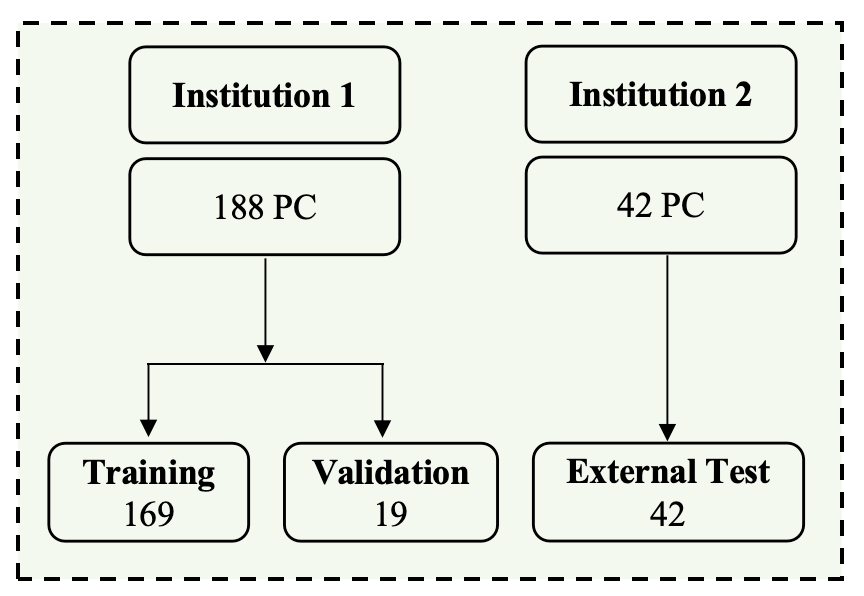


**Supplementary Figure 1. Design of data sets for POPF prediction model.**


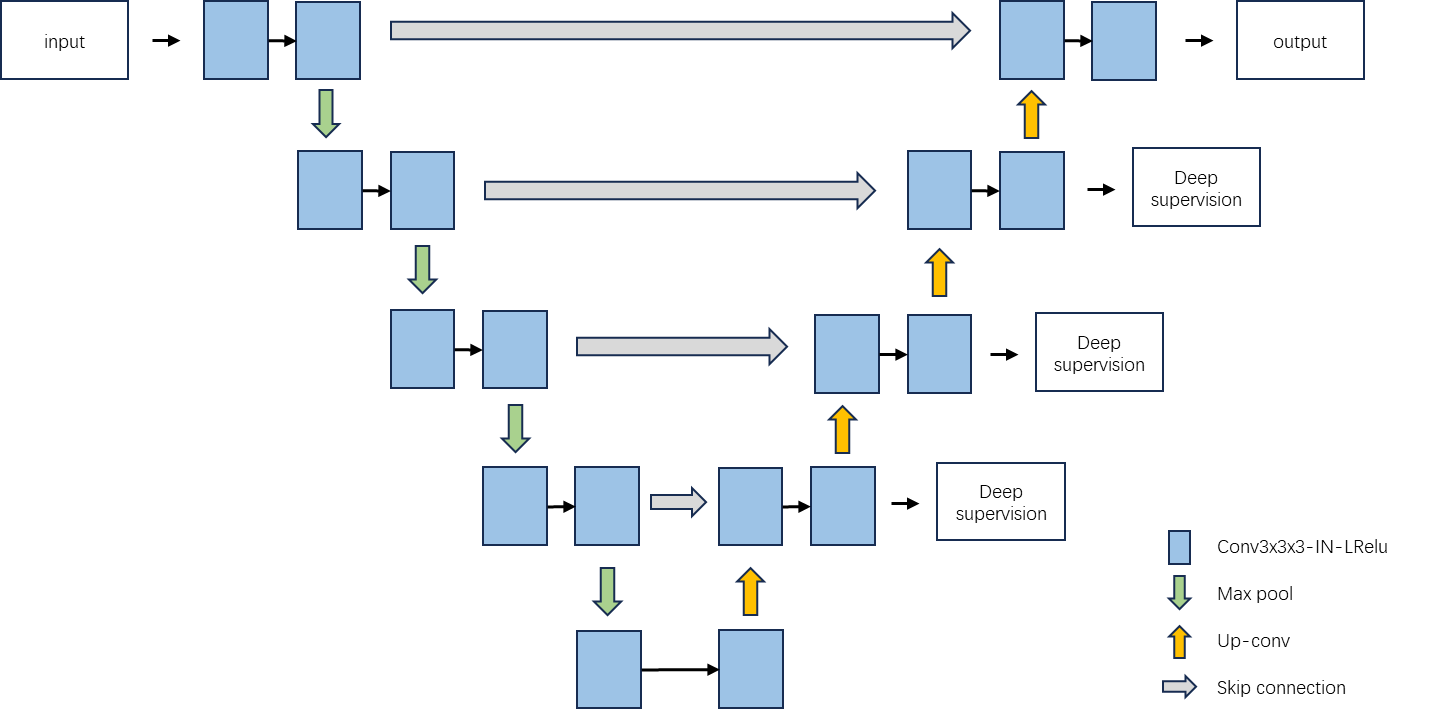


**Supplementary Figure 2. The architecture of muscle and adipose segmentation model.**

**
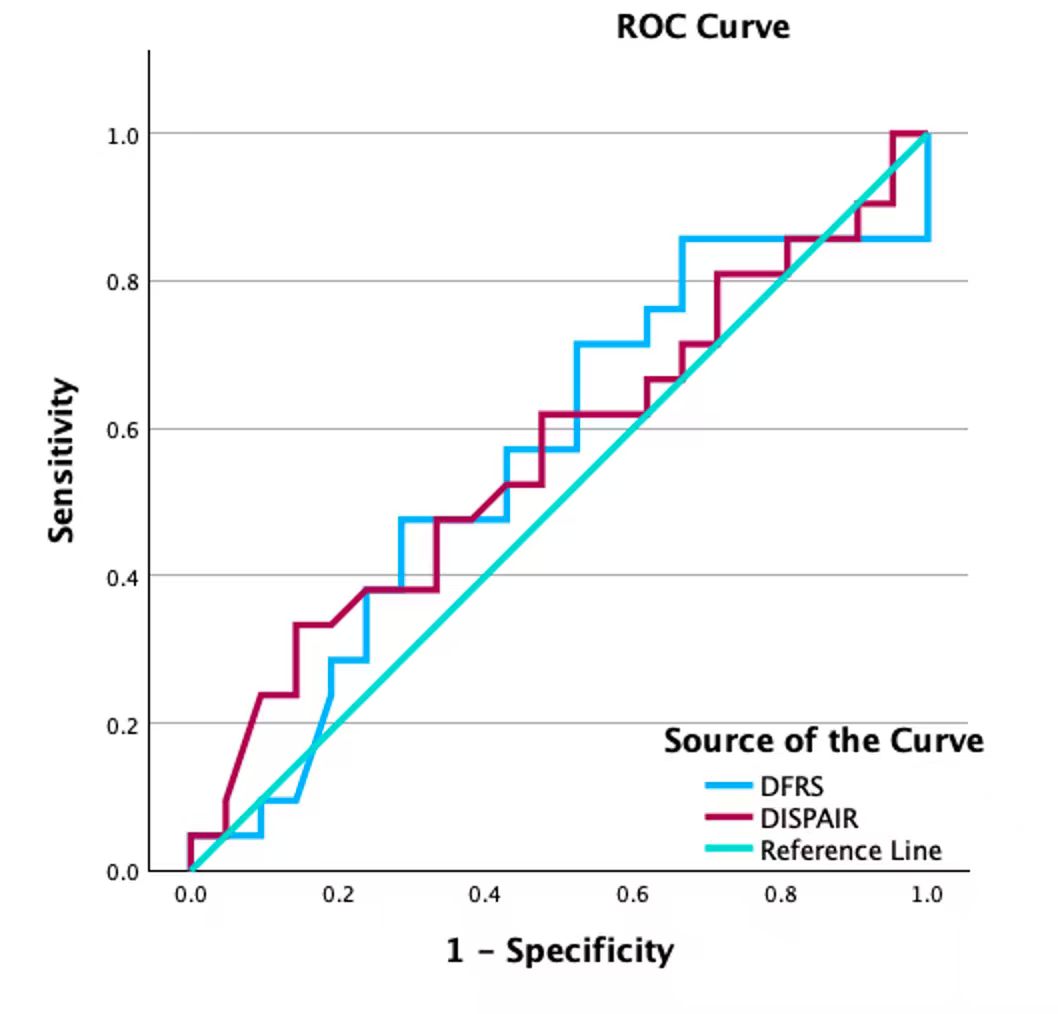
**

**Supplementary Figure 3. ROCs of D-FRS and DISPAIR models in out external testing set**
